# Special Issue “New Insights into Lactoferrin”

**DOI:** 10.3390/ijms26209891

**Published:** 2025-10-11

**Authors:** Luigi Rosa, Giusi Ianiro, Antimo Cutone

**Affiliations:** 1 Department of Public Health and Infectious Diseases, University of Rome “La Sapienza”, 00185 Rome, Italy; luigi.rosa@uniroma1.it; 2 Department of Biosciences and Territory, University of Molise, 86090 Pesche, Italy; giusy.ianiro@unimol.it

Lactoferrin (Lf) is a positively charged iron-binding glycoprotein that occupies a unique position at the interface between innate immunity and host defense [1,2]. Structurally, Lf is composed of two homologous lobes (N- and C-lobes), each subdivided into two domains, forming a cleft that tightly coordinates ferric ions and contributes to its multifunctional properties [3,4]. Secreted in exocrine fluids and stored in the secondary granules of neutrophils, it plays a central role in the protection of mucosal surfaces and in the orchestration of immune responses [5,6,7]. Its versatility is extraordinary: Lf acts not only as an iron homeostasis regulator but also as a natural antimicrobial, antiviral, antifungal, antiparasitic, antioxidant, anti-inflammatory, immunomodulatory, and anticancer agent [8,9,10,11]. Such pleiotropy is dictated by its physicochemical features, which vary according to the degree of iron saturation and are further influenced by species- and tissue-specific differences [12,13,14]. Further, Lf is able to interact with multiple cell-surface receptors, facilitating its translocation into the nucleus, where it exerts modulatory effects on gene expression [15,16,17,18]. By chelating iron, modulating cellular uptake, and attenuating the generation of reactive oxygen species, Lf reduces oxidative stress and protects tissues from damage [19,20]. This function is particularly relevant in the context of chronic and degenerative diseases, including neurodegenerative disorders such as Alzheimer’s and Parkinson’s disease, cardiovascular pathologies, and cancer, where oxidative imbalance is a hallmark of disease progression [21,22,23]. The repertoire of Lf biological activities is enormously expanded and enhanced by its ability to generate two bioactive peptides, lactoferricin and lactoferrampin, which exhibit a wide range of multifunctional properties, ranging from antibacterial, antiviral, and antitumoral [24,25,26]. This molecular plasticity has made Lf a focus of intense multidisciplinary investigation, spanning basic structural biology, cellular and molecular immunology, biotechnology, and translational medicine [27,28,29].

The articles included in this Special Issue of the *International Journal of Molecular Sciences* collectively illustrate how Lf research has moved well beyond descriptive biology into the realm of mechanism-driven and application-oriented studies. Molecular analyses now suggest that Lf can directly interact with crucial regulators of apoptosis, supporting the idea that its anticancer activity is not merely the consequence of general cytotoxicity but may derive from a targeted modulation of caspases and anti-apoptotic proteins [30]. In parallel, regenerative medicine has recognized Lf as a natural stimulator of cell proliferation and differentiation [31,32,33], including in chondrogenesis: experimental models of auricular cartilage repair demonstrate that exogenous administration of Lf promotes proliferation and differentiation of chondrocytes and fosters the maturation of elastic fibers in vivo [34]. Further, the antioxidant potential of Lf has been comprehensively reviewed, consolidating the evidence that positions it as a natural regulator of redox balance [35]. The immunomodulatory dimension of Lf also emerges with clarity: by enhancing Toll-like receptor 7 signaling in plasmacytoid dendritic cells, Lf can amplify interferon production and initiate a cascade of antiviral responses that involve both innate and adaptive immune effectors [36]. Equally relevant are the studies that explore the antimicrobial spectrum of Lf in the context of bacterial pathogens of veterinary interest. Against *Mannheimia haemolytica*, a leading cause of ruminant respiratory disease, Lf demonstrates a dual capacity: on one side, it interferes with the viability of bacteria embedded in biofilms and alters the dynamics of biofilm cycling [37]; on the other, its apo-form directly inhibits secreted metalloproteases, key virulence factors responsible for tissue damage, through mechanisms that involve direct binding near the enzymatic active site [38]. These findings provide compelling evidence that Lf, beyond its bacteriostatic and bactericidal activities, can neutralize specific bacterial effectors, highlighting its value as a non-antibiotic strategy at a time when antimicrobial resistance is a pressing global challenge [39,40,41]. Moreover, Lf has been widely recognized as a natural shaper of human microbiota, thus contributing to the maintenance of intestinal homeostasis and supporting the immune system [42,43,44]. Another dimension emphasized in this Special Issue is the nutraceutical potential of Lf [45,46]. The development of innovative formulations, such as Valpalf^®^, has shown that chemical stabilization and improved nuclear uptake can significantly enhance its biological activity, potentiating its anti-inflammatory and antioxidant effects and improving clinical outcomes in conditions characterized by inflammation and anemia [47]. This work underscores the translational trajectory of Lf, from a natural molecule to an engineered nutraceutical with documented therapeutic benefits [48,49,50]. In this respect, biotechnological modifications, such as recombinant engineering, nanoformulation, or targeted conjugation, have been explored to improve Lf stability and bioavailability [51,52,53]. Biotechnological research further strengthens this translational perspective by providing efficient systems for large-scale recombinant Lf production. A novel glucose-inducible expression platform in Pichia pastoris has been demonstrated to outperform traditional methanol-inducible systems, enabling the cost-effective and scalable generation of porcine Lf with conserved antimicrobial and anticancer properties, and even enhanced bioactivity upon enzymatic hydrolysis [54]. These advances address one of the historical limitations of Lf use, namely the availability of sufficient quantities for clinical and industrial application. Taken together, the contributions of this Special Issue portray Lf as a molecule of exceptional biological breadth, capable of addressing diverse health challenges across disciplines as different as oncology, infectious diseases, immunology, regenerative medicine, biotechnology, and nutrition. The convergence of mechanistic studies, preclinical models, novel biotechnological platforms, and translational applications confirms that Lf research is entering a mature phase, in which molecular insights are increasingly translated into tangible innovations. Looking forward, future investigations should prioritize clinical validation of preclinical findings, the integration of Lf-based nutraceuticals into therapeutic regimens, and the optimization of industrial production systems. In this way, Lf may fully realize its potential as both a natural effector of immunity and a versatile therapeutic and nutraceutical agent, capable of responding to the biomedical challenges of our time.
